# A model of learning temporal delays, representative of adaptive myelination

**DOI:** 10.1186/1471-2202-16-S1-P29

**Published:** 2015-12-18

**Authors:** Meenakshi Asokan, Karishma Chhabria, V Srinivasa Chakravarthy

**Affiliations:** 1 Department of Electrical Engineering, Indian Institute of Technology Madras, Chennai, 600036, Tamil Nadu, India; 2 Department of Biotechnology, Indian Institute of Technology Madras, Chennai, 600036, Tamil Nadu, India

## 

Learning and plasticity in the brain has been generally attributed to the synaptic activity in a neuronal network. However, recent studies [1] propose that the changes in conduction velocity of action potentials could affect the synchrony of spike arrival timings at the synapse, thereby modulating plasticity. This is attributed to adaptive myelination brought about by the oligodendrocytes (a class of glia that myelinate the axons in the central nervous system). We propose that the temporal delays in a neuronal network could be trained in addition to the training solely synaptic weights, in response to dynamic input spike patterns. These temporal delays are trained using the Spike Timing Dependent Plasticity (STDP) kernel, which is a temporally asymmetric variant of Hebbian learning. This paradigm of modeling is motivated from a study which describes that in addition to the pre-synaptic activity, oligodendrocytes can sense the post synaptic activity relayed through the astrocyte activity [2].

**Figure 1:**
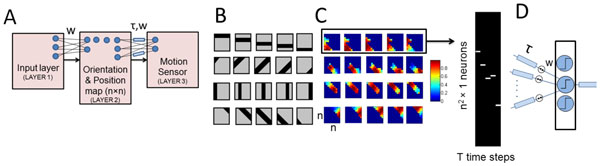
**A. Network architecture B**. Pictorial representation of the input patterns (first layer) **C**. Output of the SOM (second layer) corresponding to each input pattern **D**. Processed second layer output is fed to the third layer to train the delays (τ) and the weights (w).

The proposed model comprises of three layers (Figure 1A.); the first layer represents the input (different bar orientations and corresponding spatial locations (Figure 1B.)) to the Self Organizing Map (SOM) (second layer). For every bar orientation, a different neuron in the SOM is activated for each spatial location (Figure 1C.). This sequence of static outputs are cascaded depending on the direction of motion for each orientation and fed to the third layer as dynamic spike trains (Figure 1D.). The weights between the second and the third layer are trained by Hebbian learning and normalized after each input presentation. Furthermore, the delays are simultaneously trained using the STDP algorithm wherein the pre-synaptic spikes are input spike trains, time shifted by temporal delays. The post synaptic spikes are calculated by integrating the Post Synaptic Potentials (PSPs), for a given threshold and the neuron having the maximum amplitude of the integrated PSP is chosen as the winner. Simulation of such a network results in different neurons activated in response to motions of bars of different orientations. In contemporary neural network studies, temporal delays are typically ignored or held constant. However, the plasticity of conduction delays adds a novel dimension to the study of neural information processing. Moreover, future exploration in this domain could possibly explain the correlations of hyper and hypo synchrony of neural firing with disorders such as dyslexia and schizophrenia [3].
